# Combining contour-based and region-based in image segmentation

**DOI:** 10.12688/f1000research.140872.2

**Published:** 2023-11-16

**Authors:** Issam Dagher, Elie Abboud

**Affiliations:** 1 Computer Engineering Department, University of Balamand, Balamand, North Governorate, Lebanon

**Keywords:** Image Segmentation. Clustering. Edge detection. Colour frequencies. Texture.

## Abstract

**Background:**

This paper presents an optimized clustering approach applied to image segmentation. Accurate image segmentation impacts many fields like medical, machine vision, object detection. Applications involve tumor detection, face detection and recognition, and video surveillance.

**Methods:**

The developed approach is based on obtaining an optimum number of clusters and regions of an image. We combined Region-based and contour-based approaches. Initial rough regions are obtained using edge detection. We have used Gabor wavelets for texture classification and spatial resolutions. Color frequencies are also used to determine the number of clusters of the Fuzzy c-means (FCM) algorithm which gave an optimum number of clusters or regions.

**Results:**

We have compared our approach with other similar wavelet and clustering techniques. Our algorithm gave better values for segmentation metrics like SNR, PSNR, and MCC.

**Conclusions:**

Optimizing the number of clusters or regions has a significant effect on the performance of the image segmentation techniques. This will result in better detection and localization of the segmentation-based application.

## Introduction

Image segmentation is a fundamental step in computer vision for object recognition and classification. Despite many techniques and algorithms have been proposed, image segmentation remains one of the most challenging research. ^1^ Two explanations can be attributed to the complexity of image segmentation. The first is that image segmentation has many solutions for the problem i.e. for one image, there are many best results of segmentation. The second is because of noise, background, low signal-to-noise-ratio, and uninformed intensity. ^2^ For that, it is difficult to only suggest one image segmentation method. We can distinguish between two concepts in image segmentation: region-based and contour-based techniques.

Region-based approaches partition the image into different homogenous regions based on similarities in color, location, and texture.

Contour-based techniques start with edge detection technique followed by linking and forming the segments.

In this paper, we tried to combine both approaches. We start with the Canny edge detector. Then we form initial regions accordingly. Those regions are optimized and merged according to similarities in color, location, and texture.

### Related work

Over recent years, several techniques have been developed to segment images. Wavelet-based segmentation can be found in Ref. 3. Unsupervised image segmentation ^4^ is performed using k-means clustering. It clusters (segments) the image into different homogenous regions. In Ref. 5 Graph theory was employed using greedy decisions. Segmentation using Texture is shown in Sagiv *et al*. ^6^ Shi *et al.* ^7^ used smoothness and boundary continuity. Ren and Malik ^8^ used contours and textures. In Refs. 9 and 10 the concept of superpixels was used where the redundancy of the image can be highly decreased Superpixel methods ^11^^,^^12^ have been researched intensively using NCut, mean shift, and graph-based methods. Genetic algorithm was also employed in Ref. 13. Edge detection techniques in image segmentation is shown in Ref. 14. Maximum variance segmentation method (MVSM in Ref. 3): Segmentation is done by finding the threshold that will give the maximum value of the variance between the 2 regions. Bimodal segmentation method (BSM in Ref. 3): The segmentation process is based on finding automatically the valley and the peaks of the histogram. It is based on finding the threshold corresponding to the valley between the two peaks. Valley threshold segmentation method (VTSM in Ref. 3): This method is an extension of BSM by segmenting the image with multiple valleys and multiple peaks. Wavelet segmentation method (WSM in Ref. 3): In this method, the optimum threshold is updated according to the three-level wavelet decomposition of the histogram of the image starting from a rough value of the threshold in the large scale. Content-Adaptive Superpixel Segmentation (CAS in Ref. 15): This paper locates the features in the image corresponding to color, contour, texture, and spatial characteristics. A clustering algorithm is then used to improve the importance of each feature. SLIC (Simple Linear Iterative Clustering in Ref. 9): This algorithm generates superpixels by clustering pixels based on their color similarity and proximity in the image plane.

## Image segmentation techniques

Image segmentation is the process of dividing an image into multiple partitions. It is typically used to locate objects and change the representation of the image into something more meaningful. It is also used in multiple domains such as medical imaging, object detection, face recognition, and machine vision.

Image segmentation consists of assigning a label for every pixel in an image. Moreover, different labels have different characteristics, and the same labels share the same characteristics at some point such as color, intensity, or texture. The result of image segmentation is a set of segments that collectively cover the entire image or a set of contours extracted from the image.

Different image segmentation techniques exist like threshold-based, region growth, edge detection, and clustering methods. ^1^

### Threshold-based segmentation

Threshold segmentation ^16^ is one of the most common segmentation techniques. It splits the picture into two or multiple regions using one or multiple thresholds. The most commonly used threshold segmentation algorithm is the Otsu method, which selects optimum threshold by optimizing deviation between groups. Its downside is that it is difficult to get correct results where there is no noticeable grayscale variation or overlap between the grayscale values in the image. ^2^ Since Thresholding recognizes only the gray information of the image without taking into consideration the spatial information of the image, it is vulnerable to noise and grayscale unevenness, for that it is frequently combined with other methods.

### Region growth segmentation

The regional growth approach ^17^ is a traditional serial segmentation algorithm, and its basic concept is to use identical pixel properties together to construct a region. An arbitrary seed pixel is chosen and compared with neighboring pixels. The region is grown from the seed pixel by adding neighboring pixels that are similar, increasing the size of the region. When the expansion of one region stops, another seed pixel that doesn’t yet belong to any region is chosen and therefore the flow is repeated.

### Edge detection

Edge detection ^18^ is used to find the boundaries of objects in an image. It detects discontinuities in brightness. The most common edge detection technique is Canny edge detector.

### Clustering

Clustering ^19^ is the task of dividing the population or data points into several groups such that similar data points within the same groups are dissimilar to the data points in other groups. A common clustering algorithm is the Fuzzy C-means (FCM).

Fuzzy c-means (FCM) is a clustering method that permits one piece of data to be a member of two or more clusters. Based on the distance between the cluster center and the data point, this algorithm determines each data point’s membership in relation to each cluster center.

### Connected component algorithm

The Connected component algorithm ^20^ scans an image and groups the pixels into components dependent on pixel connectivity, i.e. all pixels in the connected component share identical pixel intensity values and are in some way connected. Until all classes have been determined, each pixel shall be labelled with a gray level or a color (color marking) according to the portion to which it has been allocated. Connected part labeling works by scanning an image, pixel-by-pixel (from top to bottom and from left to right) to identify connected pixel regions, i.e. neighboring pixel regions that share the same collection of intensity values as V.

### Texture filters: Gabor wavelets

The objective of Texture filters ^21^ is to separate the regions in an image based on their texture content. While smooth regions are characterized with a small range of values in the neighborhood around a pixel, rough texture regions are characterized by a large range of values. Gabor Wavelets are band pass filters which extract the image local important features. A convolution is done between the image and the filters in order to get texture frequency and orientation. We have used the outputs of Gabor filters with 8 orientations and 5 wavelengths.

## Methods

**Figure 1.**
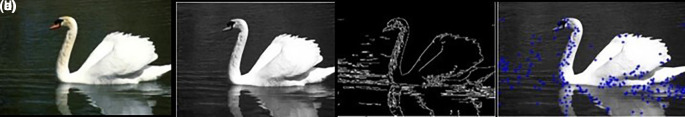
(a) Original image; (b) Grayscale image; (c) Edge image; (d) Initial set of centers.

**Figure 2.**
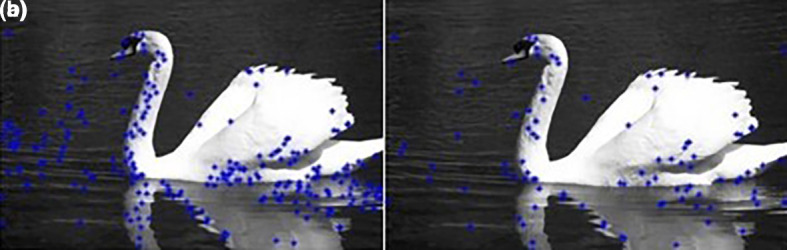
(a) Initial set of centers; (b) After the first reduction.

**Figure 3.**
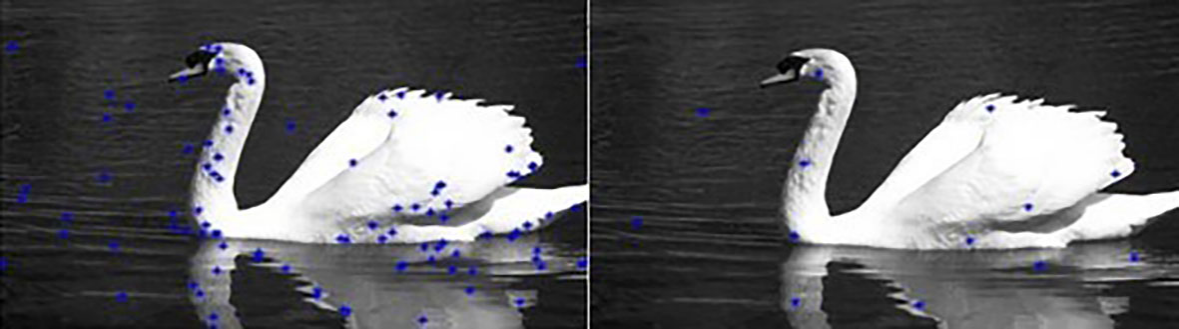
Reduction of RGB centers using FCM.

**Figure 4.**
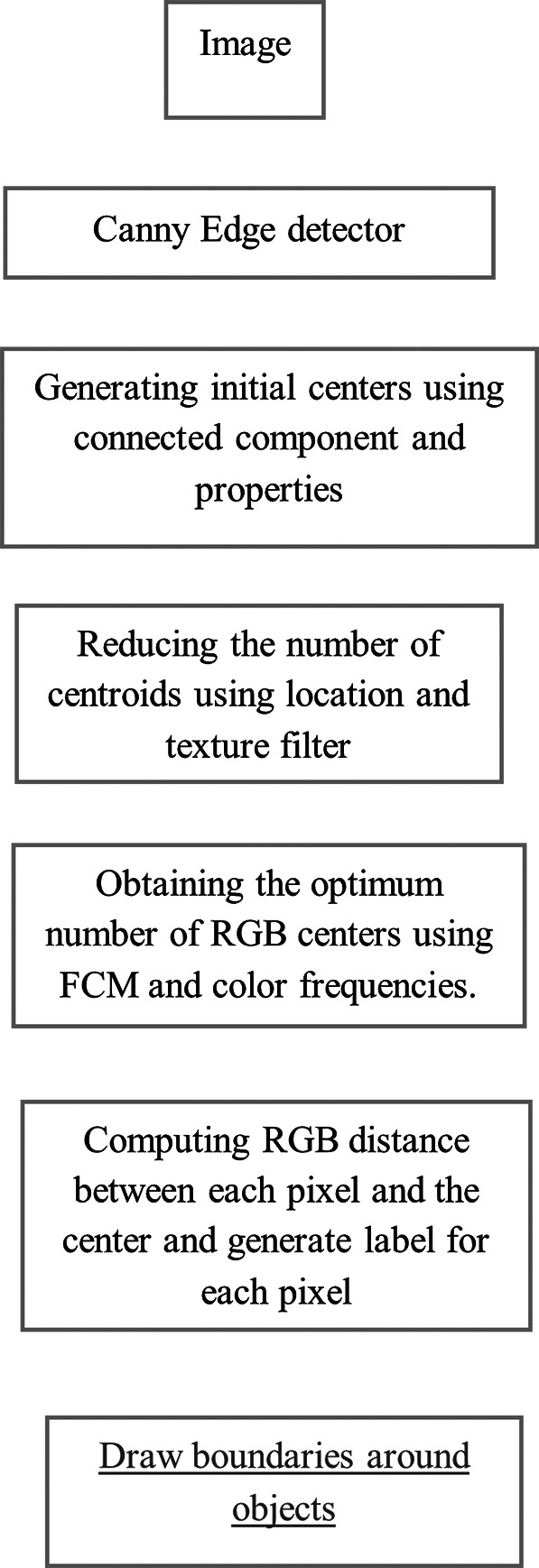
Flowchart of the proposed approach.

The proposed approach is based on obtaining an optimum number of clusters and regions of an image obtained from the Berkeley segmentation dataset. This is done using the following three consecutive steps:

I. Obtaining a good initial set of centers:
  • Apply edge detection. This is done using the canny edge detector.
  • Apply the connected component algorithm on the binary image obtained.
  • Using the labeled image, find the properties of each region.
  • Join similar regions and keep the unique ones.
  • Finally, find the center of each region.
Figure 1 illustrates the procedures of step I.
II. Reducing the number of centers
  This is done using texture filters as follows:
  • Get the feature vectors of each center using Gabor filters.
  • Merge the centers according to their Euclidian distances and the results obtained from the Gabor filters using:
    The Euclidian distance between 2 centers is given by:
$$\text{Distance}=\sqrt{{\left(\text{Xcenter}1-\text{Xcenter}2\right)}^{2}+{\left(\text{Ycenter}1-\text{Ycenter}2\right)}^{2}}$$
    Where Xcenter 1 and Ycenter 1 are the xy coordinates of the first center and Xcenter 2 and Ycenter 2 are the xy coordinates of the second center
    The features distance between 2 centers is given by:
$$\text{Feature Distance}=\sqrt{{\left(\text{Feature center}1-\text{Feature center}2\right)}^{2}}$$
  • If the 2 centers are close to each other and approximately belong to the same texture, then merge them.
Distance×Feature Distance < Threshold
    The results are shown in Figure 2.
Figure 2 shows that the number of centers was reduced from 246 to 97.
III. Apply the FCM clustering algorithm:
  It should be noted that the FCM clustering requires the specification of the number of clusters. Noting that in color image segmentation the similarity used by the FCM is based on Euclidian distance between RGB pixels, getting the number of clusters is done by using Color frequencies. The color frequencies ^22^ index is computed by three steps:
  1. All the color frequencies of the image are computed and added to an array
  2. Then, the duplications in the array are removed and unique frequencies are kept
  3. Finally, only the main colors are kept for example if there are multiple shades of a color only the main color is kept, and the other ones are removed
  The color frequencies index is equal to the size of the array and is given as an input to the FCM function. After this step is applied the number of RGB centroids is reduced from 97 centroids to only 13 ( Figure 3). Then the RGB distance is computed between each pixel and the center to determine its corresponding label.
  Our algorithm is summarized in Figure 4.

### Illustration of the algorithm

**Figure 5.**
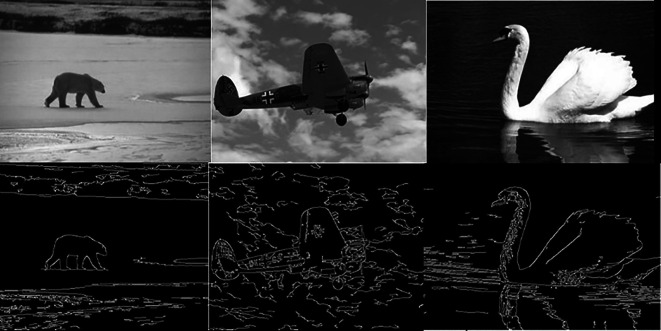
Original 3 images (Upper Row) and their Corresponding Edge images (Lower Row).

**Figure 6.**
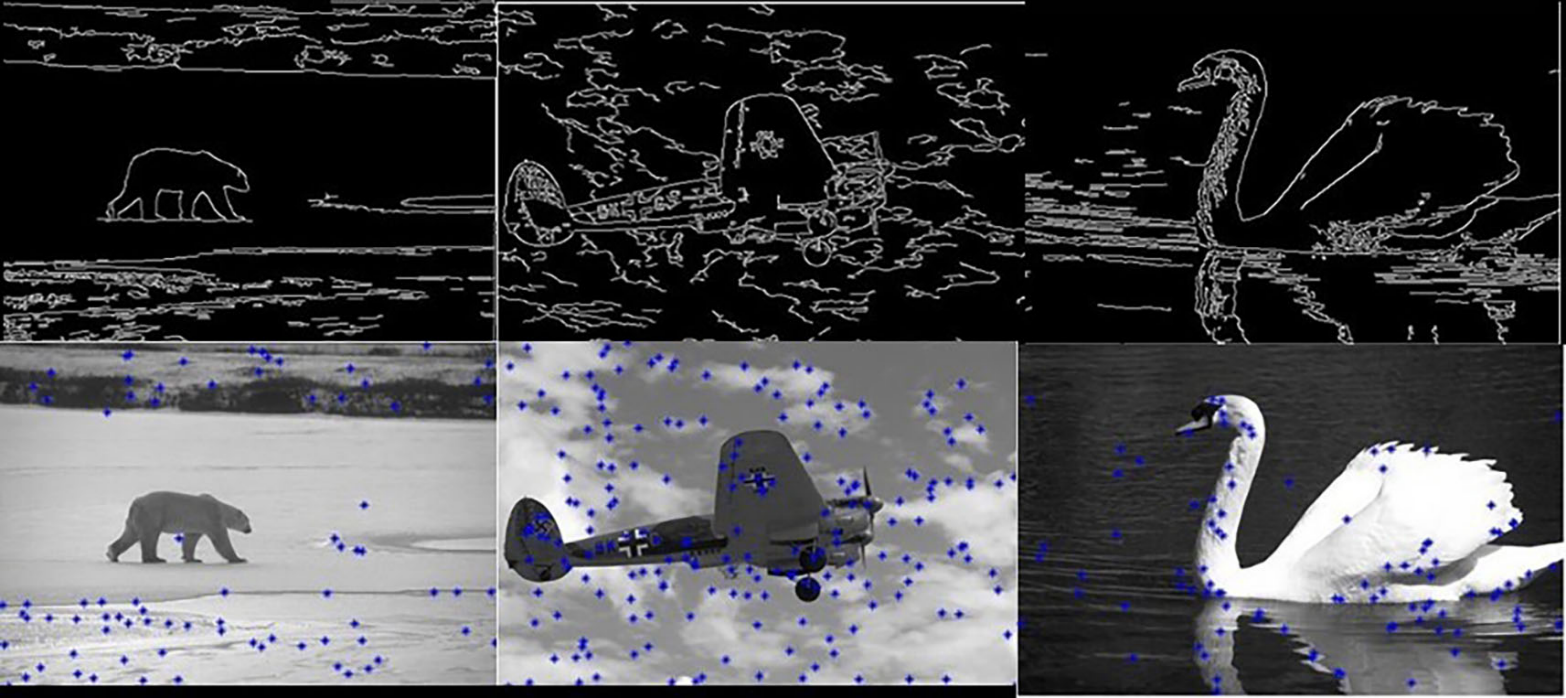
Edge images (Upper Row) and their corresponding initial centers (Lower Row).

**Figure 7.**
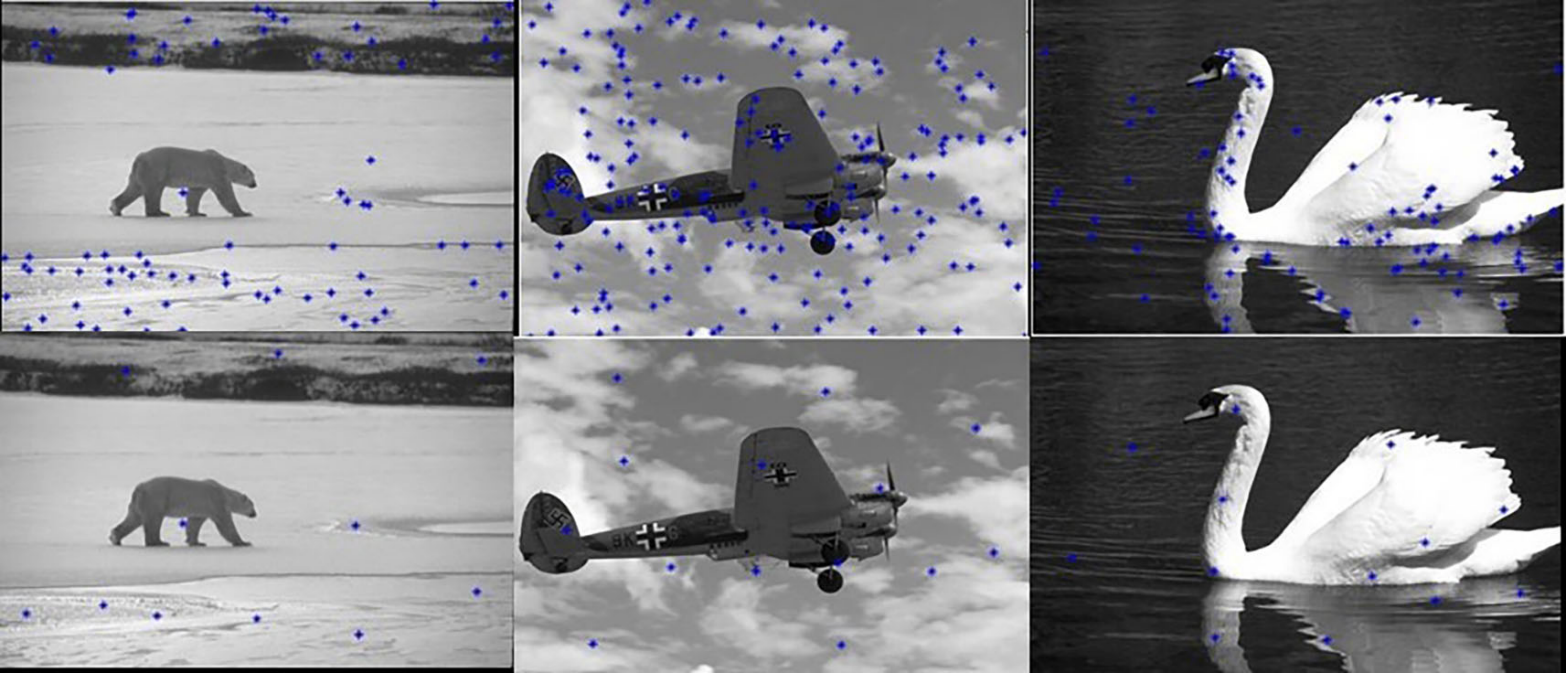
Initial set of centers (Upper row) and their corresponding optimum number of centers (Lower row).

**Figure 8.**
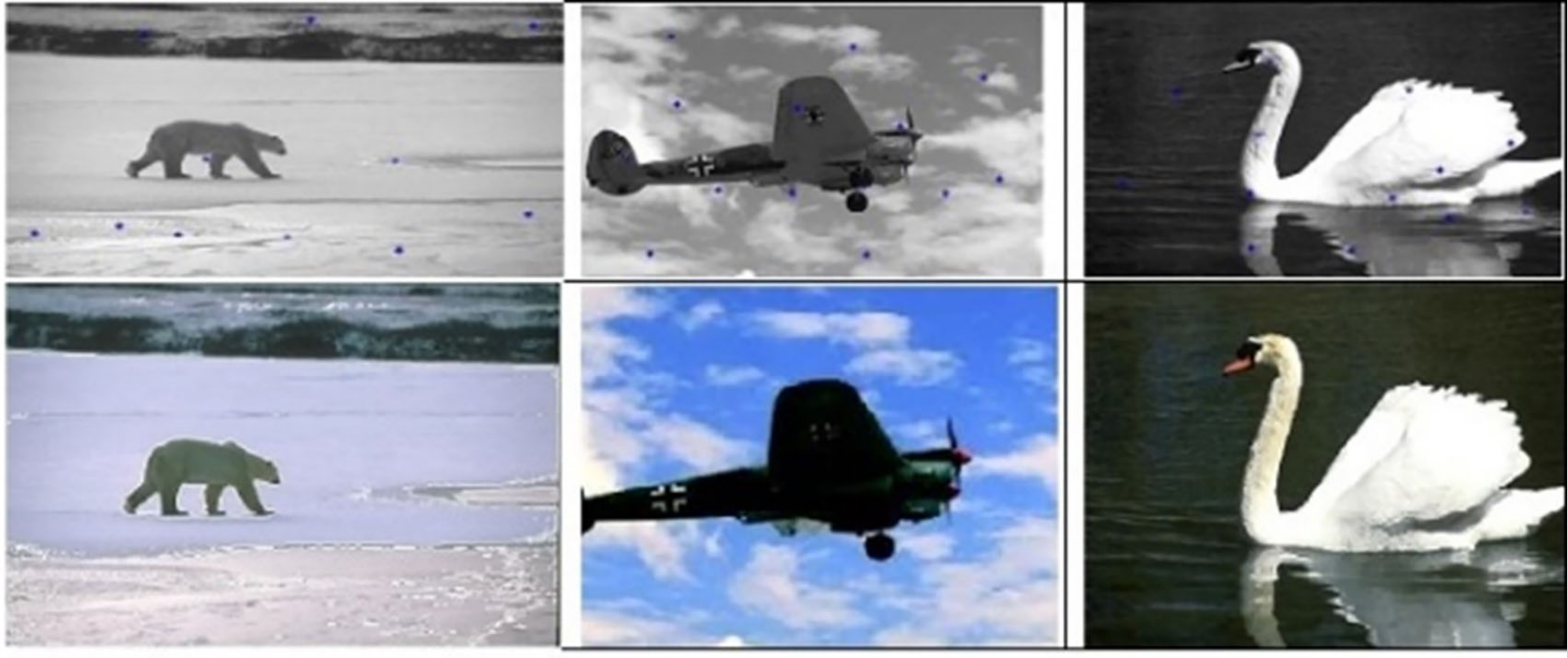
Segmented images (lower row) and their corresponding optimum number of centers (upper row).

Figure 5 shows 3 images and their edge images. Figure 6 shows the edge images and their corresponding initial set of centers. The optimum number of cluster centers is shown in Figure 7. The final image segmented images are shown in Figure 8.

## Results

### Dataset

**Figure 9.**
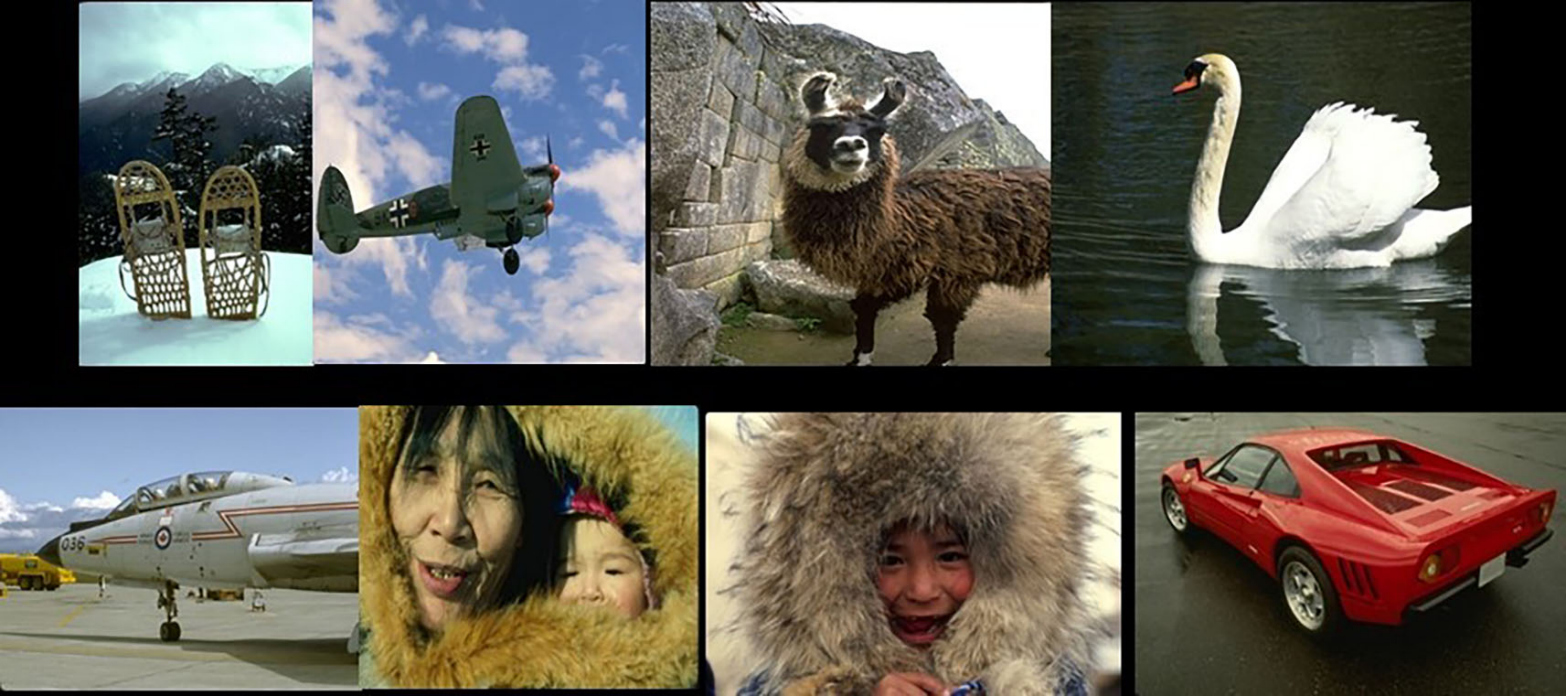
Samples from the BSD500 database.

To evaluate this work, the BSDS500 database ^23^ is chosen. It is used for most segmentation techniques. It consists of 500 images of outdoor scenes, landscapes, buildings, animals, and humans. Figure 9 shows sample images from the database.

### Segmentation metrics

The following segmentation metrics ^24^ are used to show the effectiveness of our novel approach: accuracy, F-measure, precision, MCC, dice, Jaccard, specificity. Those metrics are computed by comparing the result segmented image with the ground truth of the original image.

Given that: TP is the true positive, TN is the true negative, FN is the false negative and FP is the false positive

$$\text{Accuracy}=\frac{\mathrm{TP}+\mathrm{TN}}{\mathrm{TP}+\mathrm{FN}+\mathrm{FP}+\mathrm{TN}}.\text{Precision}=\frac{\mathrm{TP}}{\mathrm{TP}+\mathrm{FP}}.\text{F measure}=\frac{\left(\beta^{2}+1\right)\mathrm{TP}}{\left(\beta^{2}+1\right)\mathrm{TP}+\beta^{2}\mathrm{FN}+\mathrm{FP}}.$$

$$\mathrm{MCC}=\frac{\mathrm{TP}*\mathrm{TN}-\mathrm{FP}*\mathrm{FN}}{\sqrt{\left(\mathrm{TP}+\mathrm{FP}\right)\left(\mathrm{TP}+\mathrm{FN}\right)\left(\mathrm{TN}+\mathrm{FN}\right)\left(\mathrm{TN}+\mathrm{FP}\right)}}.\text{J accard}=\frac{\mathrm{TP}}{\mathrm{TP}+\mathrm{FN}+\mathrm{FP}}.\text{Dice}=\frac{2\mathrm{TP}}{2\mathrm{TP}+\mathrm{FN}+\mathrm{FP}}$$

$$\text{Specivity}=\frac{\mathrm{TN}}{\mathrm{TN}+\mathrm{FN}}.$$

### Results of proposed approach

In this section, the results of the proposed approach are compared with different methods on the same database and using the same classification metrics. For the K-means and the SLIC we have experimented with different values of K and we have chosen the value of K which gave good segmentation results. We used K=10 for the K-means and K=100 For the SLIC.

***Graphical Illustration***

**Figure 10.**
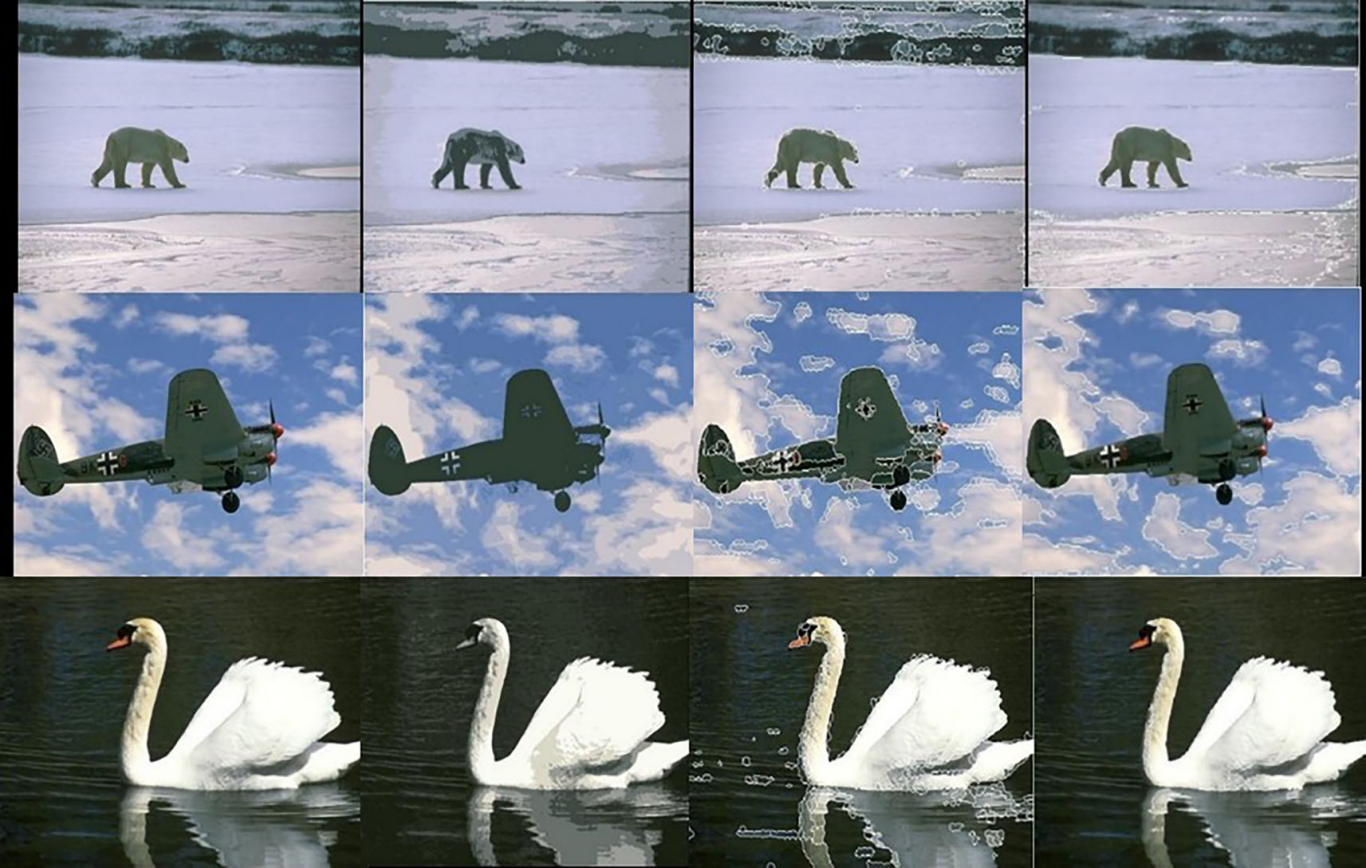
Original 3 images (First column). Results of the K-means, the SLIC, and the proposed approach in second, third, and fourth columns respectively.

The following figures illustrate the segmentation results of the Kmeans, SLIC, and our algorithm. Figure 10 shows the results obtained by the K-means, the SLIC, and our algorithm. The Figure shows the superior performance of our approach.

***Comparisons based on the Segmentation metrics***

Table 1 shows the segmentation metrics results of our algorithm compared to the K-means, the SLIC and the CAS ^15^ algorithms. The images of the BSD500 are used and the average segmentation metrics are shown in the table. Table 4.1 shows the accurate segmentation results of our algorithm compared to the others. It should be noted that our algorithm does not require a priori to specify the number of centers.

**Table 1.** Performance of the various approaches on the BSD500.

Method	Avg. Accuracy	Avg. Precision	Avg. F-measure	Avg. MCC	Avg. Jaccard	Avg. Dice	Avg. Specificity
K-means K=10	0.8842	0.8941	0.8886	0.8672	0.8696	0.8886	0. 8977
SLIC K=100	0.9296	0.9316	0.9284	0.8918	0.9111	0.9284	0.9267
CAS K=400	0.9711	0.9668	0.9454	0.9221	0.9654	0.9756	0.9775
Proposed approach	0.9841	0.9776	0.9543	0.9231	0.9732	0.9811	0.9844

**Figure 11.**
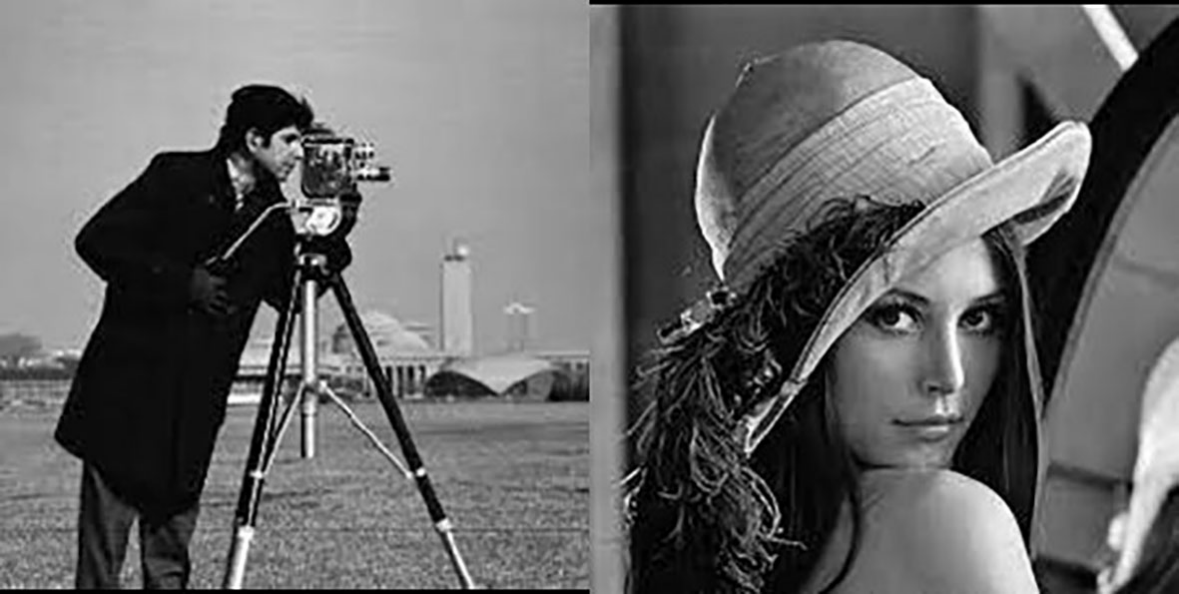
Cameraman and Lena images.

To show the effectiveness of the proposed method, we have followed the experiments done in Ref. 3 using 2 images: Lena and the Cameraman images ( Figure 11). We have used the SNR and the PSNR as verification indices. Table 2 shows the results obtained. It clearly shows the outperformance of our approach.

**Table 2.** Performance of our approach for the 2 images compared to the results obtained in Ref. 3.

Methods	SNR	PSNR
**Lena**		
MVSM	46.0638	3.5854
BSM	45.6782	3.1999
VTSM	46.1026	3.6242
WSM	48.1855	5.7071
Our	50.89	7.78
**Cameraman**		
MVSM	45.6261	3.0267
BSM	47.4184	4.819
VTSM	45.6929	3.0935
WSM	48.1859	5.5865
Our	50.76	7.88

Bigger SNR and PSNR imply better segmentation results. Our algorithm gave for the Lena image an SNR 0f 50.89 and PSNR of 7.78 which are bigger than the other 4 algorithms.

## Conclusion

Image segmentation has become an important topic in many fields like medical, machine vision, object detection. Different segmentation techniques exist. Segmentation by edge detection (based on Gradient vector). Segmentation by thresholding (based on computing the threshold from the histogram). Segmentation by clustering (FCM is used to separate the image into different clusters or regions). Segmentation by texture analysis (partitioning into regions according to their textures). Segmentation by wavelet (decomposition the image into different subbands). In this work, a new approach is proposed to improve the accuracy and performance of image segmentation. We combined Region-based and Contour-based segmentation both approaches. Edge detection, Color frequencies, and texture measures are used in developing the new algorithm. We started with Canny edge detector. Then we formed initial regions accordingly. Those regions are optimized and merged according to similarities in color, location and texture. We obtained optimum number of clusters and regions of an image. To show the effectiveness of this work, the BSDS500 database is chosen and different segmentation and clustering measures were used. The results show the improved performance of the proposed technique compared to other wavelet-based and other techniques.

It should be noted that the proposed approach is unsupervised. It cannot classify the segmented regions. For classification applications, this approach should be followed by neural networks or by directly using deep learning.

## Data Availability

All images used in this article were sourced from The Berkeley Segmentation Dataset and Benchmark (BSDS300): https://www2.eecs.berkeley.edu/Research/Projects/CS/vision/grouping/resources.html#algorithms ^23^ Zenodo. COMBINING CONTOUR-BASED AND REGION-BASED IN IMAGE SEGMENTATION. https://doi.org/10.5281/zenodo.8319898. ^25^ This project contains the following extended data:
• Code.docx (analysis code) Data are available under the terms of the Creative Commons Attribution 4.0 International license (CC-BY 4.0).
