# Supplementary material for: Combining contour-based and region-based in image segmentation
Source: F1000Res. 2024 Apr 2;12:1312. Originally published 2023 Oct 11. [Version 3] doi: 10.12688/f1000research.140872.3 (PMC11325159; doi:10.12688/f1000research.140872.3)
Supplement: Peer review history

# Reviewer report: Reviewer response for version 3

**DOI:** 10.5256/f1000research.164179.r304623

**Published:** 2024-08-14

**Authors:** Nishtha Parashar

**Affiliations:** 1 Department of Computer Science & Engineering, Amity University (Ringgold ID: 77282), Gwalior,, Madhya Pradesh, India

1. The proposed strategy needs further optimization to enhance its effectiveness.

 2. A detailed comparison of the proposed approach with existing methodologies should be added in the paper.

 3. The flow chart should be redesigned.

 4. The grammar should be rechecked to ensure that sentences follow the passive voice, improving the effectiveness of the research paper.

Is the work clearly and accurately presented and does it cite the current literature?

Partly

If applicable, is the statistical analysis and its interpretation appropriate?

Yes

Are all the source data underlying the results available to ensure full reproducibility?

Yes

Is the study design appropriate and is the work technically sound?

Partly

Are the conclusions drawn adequately supported by the results?

Partly

Are sufficient details of methods and analysis provided to allow replication by others?

Yes

Reviewer Expertise:

Image Mining, Computer Vision

I confirm that I have read this submission and believe that I have an appropriate level of expertise to confirm that it is of an acceptable scientific standard, however I have significant reservations, as outlined above.

## References

[1] : Hierarchical open-vocabulary universal image segmentation. *https://www.researchgate.net/publication/372073999_Hierarchical_Open-vocabulary_Universal_Image_Segmentation/* .2023;

[2] : A feature-weighted suppressed possibilistic fuzzy c-means clustering algorithm and its application on color image segmentation. *Expert Systems with Applications* .2024;241: 10.1016/j.eswa.2023.122270 10.1016/j.eswa.2023.122270

# Reviewer report: Reviewer response for version 3

**DOI:** 10.5256/f1000research.164179.r261908

**Published:** 2024-04-10

**Authors:** Sandra Jardim

**Affiliations:** 1 Instituto Politecnico de Tomar, Tomar, Santarém District, Portugal

All comments and suggestions were taken into consideration by the authors when reviewing the manuscript.

Is the work clearly and accurately presented and does it cite the current literature?

Partly

If applicable, is the statistical analysis and its interpretation appropriate?

Yes

Are all the source data underlying the results available to ensure full reproducibility?

Yes

Is the study design appropriate and is the work technically sound?

Partly

Are the conclusions drawn adequately supported by the results?

Partly

Are sufficient details of methods and analysis provided to allow replication by others?

Yes

Reviewer Expertise:

Machine/Deep Learning; Computational Vision; Image Processing

I confirm that I have read this submission and believe that I have an appropriate level of expertise to confirm that it is of an acceptable scientific standard.

# Reviewer report: Reviewer response for version 2

**DOI:** 10.5256/f1000research.158898.r236265

**Published:** 2024-03-07

**Authors:** Farid Garcia Lamont

**Affiliations:** 1 Universidad Autónoma del Estado de México, Texcoco, Mexico

The paper is well written and easy to follow. However, the main weakness of the paper is that the authors do not compare their results regarding related works, since they do not employ the metrics that usually are used to evaluate quantitatively the segmented images.

 The authors employ the Berkeley Segmentation Database to test their methodology. This dataset is widely employed as bechmark by several related works to test algorithms of image segmentation by color features; the database contains ground truth (images hand-segmented) of each image. So, the resulting images are compared regarding their respective ground truth using the probabilistic random index, variation information and global consistency error metrics, to evaluate quantitatively. I suggest the authors to employ these metrics; thereby, it would be easier to compare the performance of their model with respect to previous works that employ this database.

Is the work clearly and accurately presented and does it cite the current literature?

Yes

If applicable, is the statistical analysis and its interpretation appropriate?

Partly

Are all the source data underlying the results available to ensure full reproducibility?

Yes

Is the study design appropriate and is the work technically sound?

Partly

Are the conclusions drawn adequately supported by the results?

Partly

Are sufficient details of methods and analysis provided to allow replication by others?

Partly

Reviewer Expertise:

Image processing by color features.

I confirm that I have read this submission and believe that I have an appropriate level of expertise to confirm that it is of an acceptable scientific standard, however I have significant reservations, as outlined above.

# Response

**Published:** 2024-03-22

**Authors:** Issam Dagher

**Affiliations:** Computer Engineering, University of Balamand, Balamand, North Governorate, Lebanon

"The authors employ the Berkeley Segmentation Database to test their methodology. This dataset is widely employed as bechmark by several related works to test algorithms of image segmentation by color features; the database contains ground truth (images hand-segmented) of each image. So, the resulting images are compared regarding their respective ground truth using the probabilistic random index, variation information and global consistency error metrics, to evaluate quantitatively. I suggest the authors to employ these metrics; thereby, it would be easier to compare the performance of their model with respect to previous works that employ this database."

 I did more simulations using the PRI validation index.

 And I compared our algorithm with other algorithms.

 The results are shown in Table 3.

# Reviewer report: Reviewer response for version 1

**DOI:** 10.5256/f1000research.154274.r215122

**Published:** 2023-11-03

**Authors:** Sandra Jardim

**Affiliations:** 1 Instituto Politecnico de Tomar, Tomar, Santarém District, Portugal

In the manuscript “Combining contour-based and region-based in image segmentation” is presented an image segmentation framework that combines region and contour-based approaches. The work presented by the authors addresses an interesting and current subject, but it presents some flaws that I consider relevant and that, in my opinion, should be addressed and resolved.

 My comments are as follows:

- The statement "...because none of them can provide a coherent framework for achieving quick and efficient segmentation of images" has as reference a work published in 2019. Since then, many image segmentation approaches have been proposed. Indeed, in my opinion, some of them with very good results.
- The authors should consider adding a "Related work" section, where they should present and discuss the most relevant and recent image segmentation approaches.
- There are other and recent approaches like the one presented by the authors. Although the comparison made with the chosen methods is interesting, authors must compare the proposed method with similar approaches, to demonstrate the innovation of their proposal when compared to the most recent published works.
- From the middle of the first page to the beginning of the 5th, the authors only describe basic and well-known concepts/methods. I think that it can/should be resumed.
- Figure 4 - The image is not a flowchart in a standard form. The authors should consider presenting a conventional flowchart.
- Figure 8 - From the images presented it's difficult to evaluate the results. Indeed, it seems that the plane is not segmented.
- The methods identified by acronyms in Table 2 must be identified in its full description. Additionally, a reference must be given for each one.
- The conclusions are too succinct and should be rewritten in greater detail. The conclusions are too succinct and should be rewritten in greater detail. Additionally, the limitations of the proposed approach must be mentioned.

Is the work clearly and accurately presented and does it cite the current literature?

Partly

If applicable, is the statistical analysis and its interpretation appropriate?

Yes

Are all the source data underlying the results available to ensure full reproducibility?

Yes

Is the study design appropriate and is the work technically sound?

Partly

Are the conclusions drawn adequately supported by the results?

Partly

Are sufficient details of methods and analysis provided to allow replication by others?

Yes

Reviewer Expertise:

Machine/Deep Learning; Computational Vision; Image Processing

I confirm that I have read this submission and believe that I have an appropriate level of expertise to confirm that it is of an acceptable scientific standard, however I have significant reservations, as outlined above.

# Response

**Published:** 2023-11-13

**Authors:** Issam Dagher

**Affiliations:** Computer Engineering, University of Balamand, Balamand, North Governorate, Lebanon

1. As you suggested I removed the statement "...because none of them can provide a coherent framework for achieving quick and efficient segmentation of images"

 2. I added a Related work paragraph:

 3. I compared my approach with similar approaches:

- Content-Adaptive Superpixel Segmentation.
- Linear Iterative Clustering.
- k-means
- Wavelet.
- MVSM          
- BSM  
- VTSM

4. The basic and well known methods are summarized.

 5. I changed  the flowchart in Figure 4.

 6. I changed Figure 8.

 7. I explained all the methods in the Related work section.

 8. I included more details in the Conclusion section.

 I thank the reviewer for putting his time in improving the paper.
